# Reducing Antibiotic Prescriptions for Urinary Tract Infection in Nursing Homes Using a Complex Tailored Intervention Targeting Nursing Home Staff: Protocol for a Cluster Randomized Controlled Trial

**DOI:** 10.2196/17710

**Published:** 2020-05-08

**Authors:** Sif Helene Arnold, Jette Nygaard Jensen, Marius Brostrøm Kousgaard, Volkert Siersma, Lars Bjerrum, Anne Holm

**Affiliations:** 1 The Section of General Practice and Research Unit for General Practice Department of Public Health University of Copenhagen Copenhagen Denmark; 2 Department of Clinical Microbiology Herlev and Gentofte Hospital University of Copenhagen Herlev Denmark

**Keywords:** urinary tract infection, nursing home, antibiotics, antibiotic resistance, drug prescription, communication, communication barriers, interprofessional relationship, elderly

## Abstract

**Background:**

Urinary tract infection (UTI) is the most common reason for antibiotic prescription in nursing homes. Overprescription causes antibiotic-related harms in those who are treated and others residing within the nursing home. The diagnostic process in nursing homes is complicated with both challenging issues related to the elderly population and the nursing home setting. A physician rarely visits a nursing home for suspected UTI. Consequently, the knowledge of UTI and communication skills of staff influence the diagnosis.

**Objective:**

The objective of this study is to describe a cluster randomized controlled trial with a tailored complex intervention for improving the knowledge of UTI and communication skills of nursing home staff in order to decrease the number of antibiotic prescriptions for UTI in nursing home residents, without changing hospitalization and mortality.

**Methods:**

The study describes an open-label cluster randomized controlled trial with two parallel groups and a 1:1 allocation ratio. Twenty-two eligible nursing homes are sampled from the Capital Region of Denmark, corresponding to 1274 nursing home residents. The intervention group receives a dialogue tool, and all nursing home staff attend a workshop on UTI. The main outcomes of the study are the antibiotic prescription rate for UTI, all-cause hospitalization, all-cause mortality, and suspected UTI during the trial period.

**Results:**

The trial ended in April 2019. Data have been collected and are being analyzed. We expect the results of the trial to be published in a peer-reviewed journal in the fall of 2020.

**Conclusions:**

The greatest strengths of this study are the randomized design, tailored development of the intervention, and access to medical records. The potential limitations are the hierarchy in the prescription process, Hawthorne effect, and biased access to data on signs and symptoms through a UTI diary. The results of this trial could offer a strategy to overcome some of the challenges of increased antibiotic resistance and could have implications in terms of how to handle cases of suspected UTI.

**Trial Registration:**

ClinicalTrials.gov NCT03715062; https://clinicaltrials.gov/ct2/show/NCT03715062

**International Registered Report Identifier (IRRID):**

DERR1-10.2196/17710

## Introduction

Urinary tract infection (UTI) is the most common reason for antibiotic prescription in elderly individuals living in nursing homes in Europe, and many of these prescriptions are considered inappropriate [1-3]. Overall, excessive use of antibiotics causes antibiotic-related harms, such as selection of resistant bacteria, in those who are treated and others residing within the same nursing home [4,5]. The excessive use of antibiotics for suspected UTI could be connected with the interplay between properties specific to the elderly population and the nursing home setting.

Two dominant factors associated with elderly individuals living in nursing homes contribute to overtreatment. First, it is widely accepted by nursing home staff that unspecific changes in behavior indicate UTI [6]. However, guidelines recommend that unspecific symptoms in elderly individuals should not be treated, and the link to UTI remains debated [7-9]. Second, health professionals frequently use urinary tests as screening tools for UTI [10]. As asymptomatic bacteriuria is common in nursing home residents, these tests are frequently positive [11]. Asymptomatic bacteriuria should not be treated with antibiotics, and it is commonly confused with UTI [11,12]. Therefore, urinary testing becomes a driver for overtreatment [13,14].

In the Capital Region of Denmark, most nursing homes are public, but private homes also follow municipality regulations, making labor coverage similar. A home with 60 residents employs 2 to 3 nurses and 20 to 25 health care helpers and health care assistants (in Danish, “SOSU-hjælper” and “SOSU-assistent,” respectively). Health care helpers and assistants attend to the residents’ everyday needs. Only if a resident appears unwell, a nurse gets involved. When UTI is suspected, the staff usually contacts the residents’ general practitioner (GP), the affiliated nursing home physician, or the out-of-hour service. In addition, physicians often prescribe antibiotics for UTI without directly evaluating the patients themselves, and therefore, rely on the clinical history provided by the nursing home staff [15,16].

Owing to the interaction between the properties of the elderly population and the nursing home setting, diagnosis and treatment are directly influenced by the knowledge and communication skills of the staff [17]. If nursing home staff do not have sufficient knowledge about UTI in elderly individuals or fail to communicate important clinical observations, unnecessary antibiotic prescription could be exacerbated [18]. Although the responsibilities of the staff vary, health care helpers, assistants, and nurses are engaged in the diagnostic process and treatment of elderly residents, and therefore, all staff members should be targeted in an intervention. Some antibiotic stewardship programs have improved the knowledge of UTI and have implemented decision algorithms and communication tools to reduce inappropriate antibiotic prescription for UTI, but none of the programs have randomized and tailored the intervention to the setting, in addition to targeting all groups of nursing home staff [19,20].

We hypothesize that a tailored complex intervention improving the knowledge of UTI and communication skills in nursing home staff will decrease the number of antibiotic prescriptions for UTI in residents, without changing hospitalization and mortality. The aim of this study is to describe a protocol for measuring the effect of this intervention.

## Methods

### Trial Details

The protocol describes an open-label cluster randomized controlled trial with two parallel groups and a 1:1 allocation ratio. Originally, we planned recruitment and allocation of nursing homes to be completed by the end of 2017. We aimed to complete the planning of the start-up phase (ie, the tailoring process of the intervention and scheduling of the workshops) by the summer of 2018 and finish the start-up phase by the fall of 2018. The trial was planned to last for 4 months. Data collection, analysis, and dissemination were to commence in the spring of 2019 and end in the spring of 2020.

### Recruitment

There is no central registry of nursing homes in Denmark; therefore, we use a convenience sample method. We seek volunteers through the network of hygiene nurses in the Capital Region of Denmark, who organize local meetings and conferences and have direct contact with key hygiene personnel at nursing homes.

#### Eligibility Criteria

Nursing homes enrolling in the trial should not participate in other UTI projects during the trial period. Nursing homes should be situated in the Capital Region of Denmark. They should have common areas with attending staff 24 hours a day. The living spaces for residents with dementia are included, but living spaces for other special needs are not included. Nursing home eligibility screening is to be completed during the recruitment period. Residents should be over 65 years of age and should permanently occupy a living space at an eligible home. According to Danish law, this study is a communication study. Therefore, residents meeting the eligibility criteria are included in the study, unless they or their legal guardians decline access to health information for the trial. Residents’ eligibility screening is to be performed during collection of informed consent permitting sharing of health care data.

### Intervention

The primary components are a dialogue tool and a workshop. They target all staff members who have nursing responsibilities in the intervention group. The supporting components relate to communication with stakeholders (Table 1).

**Table 1:** Primary and supporting components of the intervention.

Components		Intervention	Control
**Primary components**			
	Dialogue tool	+	−
	Workshop	+	−
**Supporting components**			
	Letter to nursing home physicians	+	−
	Letter to all staff members	+	−
	Poster for staff members	+	−
	Letter to the liaison officer in the municipality	+	+
	Letter to local coordinators	+	+
	Letter to residents and relatives	+	+
	Poster for residents and relatives	+	+

Each component of the intervention is described below, and the components received by the intervention group and those received by both the intervention and control groups are presented. Examples of the components are provided in Multimedia Appendices 1-9.

#### Interventions Received by the Intervention Group

##### Dialogue Tool

The dialogue tool consists of a reflection and a communication section. The reflection section has three parts. First, a form that enables systematic gathering of signs and symptoms exhibited by the resident. Second, a flowchart to determine if UTI is likely or unlikely. These two parts are based on the revised Loeb Minimal Criteria for ordering urinary culture [21]. Third, four questions that are designed for the staff to be able to reflect on the next actionable step. The communication section uses the communication concept called ISBAR (Identification, Situation, Background, Assessment, and Recommendation) that has previously been used in combination with a decision-making aid for UTI prescription [22]. ISBAR is used to communicate clinical information accurately among health care professionals (between staff and the prescribing physician in this case) [23].

The tool is tailored through an iterative process that involves focus groups with stakeholders. Whenever UTI is suspected, a paper copy of the tool will be available for use at the staff office. If any adverse events occur because of the tool, the local coordinators will contact the principal investigator (PI). At least two members of the study group will evaluate the main cause of the adverse event. If the tool is deemed responsible, the entire group is gathered to determine if the trial should be terminated.

##### Workshop

The tool is introduced to the staff in a 75-minute workshop. At each nursing home, we aim to include as many staff members as possible within three separate workshops. The PI will facilitate all workshops. The focus of the workshop is threefold. First, the staff members learn the distinction between UTI and asymptomatic bacteriuria. Consequently, diagnostic caveats regarding urinary testing, odor, and urine clarity will be discussed. Second, an approach to evaluate unspecific symptoms is discussed. This approach specifically considers the importance of excluding other reasons for the observed signs and symptoms. Finally, the staff members receive training on how to use the tool for test cases.

##### Letter to Nursing Home Physicians

All nursing home physicians in the intervention group receive letters that include an open invitation to participate in the workshop, information about the trial, and contact details of the study group. The letters are emailed to the nursing home physicians.

##### Letter to Staff Members

All staff members receive letters that describe the trial and their role from the management of the home and the PI.

##### Poster for Staff Members

Posters are provided in each home to remind the staff when and how to use the tool. The posters also direct the staff to local coordinators for questions. Posters are visible at staff offices.

#### Interventions Received by Both Groups

##### Letter to the General Practitioner Liaison Officer in the Municipality

In 2016, the state, the regions, the municipalities, and the General Practitioners’ Organization in Denmark agreed to employ affiliated nursing home physicians in nursing homes [24]. By 2019, the ambition was that a physician should make regular rounds at each home. However, owing to the shortage of physicians, not all homes are covered yet. Moreover, although nursing homes have affiliated physicians, some residents continue with their usual GP. Therefore, we inform nursing home physicians and GPs in the participating municipalities about the trial. In all municipalities with participating homes, we ask the GP liaison officer (“praksiskonsulent” in Danish) or another physician in the GP network to distribute a short letter informing all GPs about the trial.

##### Letter to Local Coordinators

Each home appoints a local coordinator. There are no prespecified eligibility criteria. This pragmatic approach is adopted owing to the heterogeneity of the internal organization of the home. Local coordinators in each arm receive letters describing the trial and the UTI diary.

##### Letter to Residents and Relatives

Residents and relatives are informed about the trial through letters posted in the nursing home newspaper or website containing a description of the trial and contact information of research group members.

##### Poster for Residents and Relatives

Posters containing information about the trial are prepared for residents and relatives. They are visible in common areas.

To prevent dropout, unedited trial data are returned to the homes when data collection is complete. Optionally, the PI can facilitate workshops in the control group after evaluation.

### Data Collection

In this trial, data are collected from three different sources as presented below.

#### Background Information About Nursing Homes

Background information about nursing homes is collected from the management during the enrollment period and includes the following:

1. number of living spaces for residents
2. number of livings spaces designated for normal care needs, dementia, and psychiatry
3. owner status of the home (public/private)
4. availability of dipstick (yes/no)
5. affiliated physician (yes/no)

#### Data From Nursing Home Medical Records of Residents

Collection of background information on residents commences during the trial and is collected from the nursing home medical records. The following information is obtained:

1. social security number
2. use of catheter (yes/no), and if yes, type of catheter (indwelling catheter, intermittent clean catheter, intermittent sterile catheter, or suprapubic catheter)
3. use of incontinence aids (eg, diapers and condom catheter) (yes/no)
4. mobility status (bedridden, wheelchair bound, or walking)
5. capability of providing informed consent to share health care data (yes/no)
6. treatment for diabetes (yes/no), and if yes, the type of treatment (generic drug name)
7. number of acute and prophylactic treatments for UTI (lower, upper, and urosepsis) 1 year prior to the trial
8. prophylactic treatment for UTI (yes/no), and if yes, the type of treatment (generic drug name)

Data on prescriptions for UTI, all-cause hospitalization, and all-cause mortality during the trial period are also retrospectively registered. With regard to antibiotic prescriptions for UTI, the following information is obtained:

1. generic drug name
2. start date (yyyymmdd)
3. duration of treatment (number of days)
4. strength, dosage, and frequency (mg per tablet, number of tablets per dosage, and number of doses per day)
5. prescriber background (primary physician, out-of-hour service, or hospital physician)
6. indication (curative/prophylactic UTI)

With regard to all-cause hospitalization, the following information is obtained: date of hospitalization (yyyymmdd); date of discharge (yyyymmdd); and suspected cause of hospitalization according to staff. With regard to all-cause mortality, the following information is obtained: date of death (yyyymmdd).

#### Urinary Tract Infection Diary

During the trial, local coordinators complete a diary for each resident with suspected UTI during an 8-day period. The diary is introduced to local coordinators at the nursing home during a 30-minute meeting with the PI. The diary includes the following:

1. Signs and symptoms on days 1-8
  - new urinary symptoms [dysuria, incontinence, urge, frequency, lower back pain, gross hematuria, shaking chills, suprapubic pain, and none of these]
  - other new severe symptoms [severe back pain, rigors, delirium, and none of these]
  - new onset signs of other infectious diseases [respiratory, gastrointestinal, skin, and none of these]
  - other new observations [malodorous urine, unclear urine, unspecific symptoms, and none of these]
  - temperature [degrees Celsius]
  - blood pressure [systolic/diastolic]
  - pulse [beats per minute]
  - urinary dipstick result [positive or negative for nitrite, leucocytes, and blood]
2. Events on days 1-8
  - increased observation/triage [yes/no]
  - preventive measures [yes/no]
  - dipstick test [yes/no]
  - physician contacted [yes/no]
  - urinary sample [yes/no]
  - change of catheter [yes/no]
  - antibiotic [yes/no]
  - change of antibiotic [yes/no]
  - antibiotic discontinued [yes/no]
  - result of urine culture [positive/negative/unknown/not done]
3. Prescriptions for UTI on days 1-8
  - generic drug name
  - start date [yyyymmdd]
  - duration of treatment [number of days]
  - strength, dosage, and frequency [mg per tablet, number of tablets per dosage, and number of doses per day]
4. Hospitalization on days 1-8 (day of hospitalization)
5. Death on days 1-8 (day of death)

The UTI diaries are collected once during the trial period and once after the trial has ended. Examples of UTI diaries are presented in Multimedia Appendices 10 and 11.

### Outcomes

The primary outcome is the number of antibiotic prescriptions for acute UTI per resident in 4 months following contact between the staff and a prescriber. Antibiotics prescribed during hospital visits are not included, because prescriptions by hospital physicians are independent of those by nursing home staff. Prophylactic treatments are also excluded.

The secondary outcomes are as follows: number of all-cause hospitalizations per resident in 4 months; number of all-cause deaths per resident in 4 months; and comparison of suspected UTI during the trial. Groups are compared with regard to the numbers of acute antibiotic treatments for suspected UTI, antibiotic treatments for urinary tract symptoms, UTI-related hospitalizations, and UTI-related deaths.

### Data Management

Data collected from medical records and diary entries are subjected to double data entry. A data manager performs merging, anonymization, and range checks. Trial data are stored in accordance with the data policy of the University of Copenhagen [25]. Data will be saved for 5 years after publication of the results.

### Sample Size

Our primary outcome involves clustered count data, and we determined a minimum sample size according to the method for clustered Poisson regression [26]. In Denmark, the number of prescriptions for UTI in 2015 was 90 per 100 persons among those aged above 80 years [27]. Assuming no seasonal variation and generalizability to residents, the average number of prescriptions per resident during a 4-month period is 0.3. We assume that an average nursing home accommodates 60 residents. The intraclass correlation coefficient (ICC) of antibiotic prescription in nursing homes included in similar studies varies between 0.04 and 0.17 [28-30]; the higher estimates are based on the general population and prescriptions for respiratory tract conditions, whereas the lower estimates are based on nursing home data on prescriptions for UTI. With a conservative estimate of ICC of 0.07 and a significance level of 5%, we will have a power of 80% to detect a 50% decrease in the antibiotic prescription rate for the intervention group (from 0.30 to 0.15 prescriptions per resident in 4 months). This estimation is based on the inclusion of at least 11 nursing homes and 637 residents in each arm.

### Sequence Generation, Blinding, and Statistical Analysis

The study statistician randomly assigns the nursing homes to the two study arms with a 1:1 computer generated randomization schedule stratified by municipality. Owing to the nature of the intervention, the trial is open labelled. The statistical analysis is blinded. Primary and secondary outcomes are analyzed using a Poisson regression model and generalized estimating equation.

### Ethical Approval and Sharing of Health Care Information

Because the study is not a health science project as defined in the Danish Committee Act § 2, the Research Ethics Committee of the Capital Region of Denmark has waived the need for full ethical approval (journal no: 17013412). The Danish Patient Safety Authority has approved data collection for those residents unable to participate in informed consent for sharing of their health care information (journal no: 3-3013-2409/1 and amendment no: 3-3013-2704/1). We have collected informed consent from the rest of the residents from the start-up phase until the data collection phase. The study has been reported to the Danish Data Protection Agency.

## Results

**Figure 1:**
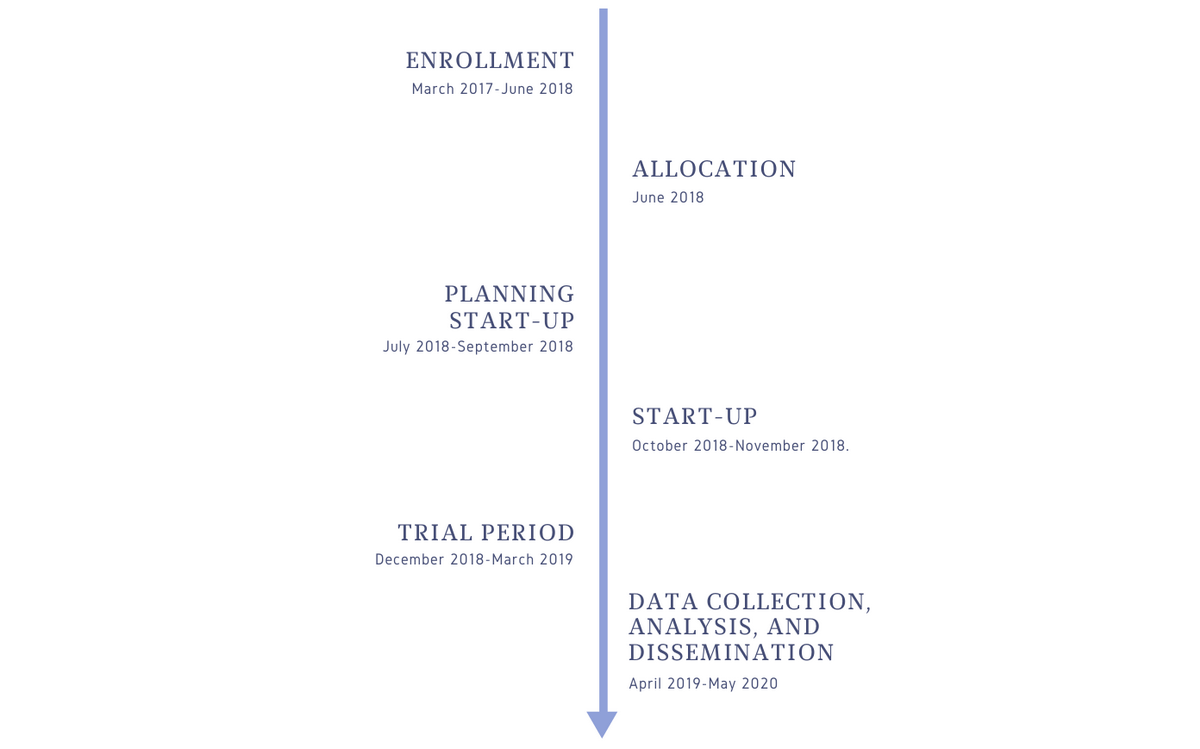
Preliminary schedule of enrollment, interventions, and assessments (February 2020).

The timeline of the trial deviated slightly from the original plan. Particularly, enrollment and allocation were completed in June 2018. The trial ended at the end of March 2019; data have been collected and are being analyzed. We expect the results of the trial to be published in a peer-reviewed journal in the fall of 2020. Figure 1 shows the schedule of enrollment, interventions, and assessments.

The original idea for the intervention was to provide nursing home staff with a biomarker for severe infection (C-reactive protein as a point-of-care test) in addition to the dialogue tool. Moreover, the primary outcome was originally appropriate prescription for UTI, but this was changed to number of prescriptions for UTI. The changes had been decided prior to registration at ClinicalTrials.gov.

## Discussion

The aim of this study is to describe a protocol for evaluating the effects on antibiotic prescription, hospitalization, and death of a tailored complex intervention to improve the knowledge of UTI and communication skills among staff caring for residents with suspected UTI. The dialogue tool is based on a decision aid and a communication tool tested in clinical trials that have previously reduced antibiotic prescriptions [21,22]. The trial was performed from December 2018 through March 2019, and the results are expected to be submitted for publication in the fall of 2020.

We apply a tailoring process to develop the intervention and decrease barriers to implementation. In order to facilitate the collection of data and to tailor the intervention process to nursing homes, the research group decided to modify the original project plan slightly. This strategy is recommended to increase the impact of complex interventions [31]. We primarily target UTI knowledge and communication skills among all staff members, which differs from the approach in previous trials [20]. Because off-site prescription in nursing homes is common, staff members collect the information the physician receives and influence the diagnosis [17]. Hence, focusing on this group may prove to be an impactful strategy. We acknowledge the hierarchy in the prescription process. If prescribers continue with inappropriate prescription for asymptomatic bacteriuria despite improved quality of information, it may overrule the effect of the intervention. To avoid this, we inform all GPs in participating municipalities about the trial and invite nursing home physicians to participate in the workshop.

The greatest strength of the study is the randomized design. On the other hand, blinding can only be introduced in the data analysis. Owing to the format of the intervention, the open-label design is indispensable but introduces the control group to the potential risks of contamination and selection bias. This bias cannot influence the primary outcome, because it is obtained from the nursing home medical records. The medical record extracts information on prescribed drugs from “Fælles Medicin Kort,” which registers all prescriptions in the past 2 years [32]. Thus, another strength of using medical records is that if an indication is unclear, background information about the prescription decision is accessible. Finally, the PI facilitates all workshops and all introductions to the UTI diary. This ensures a high degree of homogeneity in the trial start-up for all included nursing homes.

The trial is pragmatic. First, the eligibility criteria for both homes and individual residents are broad, which may result in a heterogeneous population. Second, the implementation of the intervention is left to the individual homes after completion of the workshop. Third, during the trial period, the homes are visited only once by the PI, unless they initiate contact. As the homes differ in organizational structure, a uniform and strict design was not feasible.

Nursing home medical records only include information about all-cause hospitalization and all-cause mortality. The drawback is that the information may be unrelated to UTI and that a relevant change may be diluted. We gain access to the social security numbers of residents, thereby permitting a long-term follow-up of residents through national registries. This could reveal any indirect effects on hospitalization or death. However, it is beyond the scope of this study.

Because of the Hawthorne effect, participating in a trial poses a challenge when evaluating the effect of the intervention [33]. Control groups commonly experience a behavioral change with knowledge of trial participation alone [34]. In this trial, the stakeholders in the control group are informed about the trial in general terms and both intervention and control groups have local coordinators, who complete the UTI diary. Thus, some impact of the trial design on antibiotic prescription in the control group is likely.

The use of a UTI diary may induce diagnostic bias. The diary is used for suspected UTI in both groups, but staff in the intervention group may be more alert to the discovery of potential UTI. There is a risk that staff members in the intervention group complete more diaries than staff members in the control group. The diary is extensive, and both groups may find it too bothersome to complete. This is why the research group changed the primary outcome from appropriate prescriptions to number of prescriptions, which can be found in the medical records. Nevertheless, a UTI diary is the best way to capture important clinical data about patients. Among other things, the diary includes information about signs and symptoms, which are rarely reported in medical records.

The problem of excessive and inappropriate antibiotic use is common in nursing homes. The study offers a strategy to tackle the challenges of increased antibiotic resistance and has implications in terms of handling suspected UTI. Some retailoring should be expected when transferring this intervention to an alternate setting.

In conclusion, this trial will test the hypothesis that a tailored complex intervention targeting the knowledge of UTI and communication skills of nursing home staff will improve antibiotic prescription for suspected UTI in residents. The results may provide new insights and prove to be an important addition to the current strategies to limit superfluous antibiotic treatment for suspected UTI in nursing homes.

## Appendix

Multimedia Appendix 1 Reflection section of the dialogue tool.

Multimedia Appendix 2 Communication section of the dialogue tool.

Multimedia Appendix 3 Letter to nursing home physicians.

Multimedia Appendix 4 Letter to all staff members.

Multimedia Appendix 5 Poster for staff members.

Multimedia Appendix 6 Letter to the liaison officer in the municipality.

Multimedia Appendix 7 Letter to local coordinators.

Multimedia Appendix 8 Letter to residents and relatives.

Multimedia Appendix 9 Poster for residents and relatives.

Multimedia Appendix 10 Urinary tract infection diary day 1.

Multimedia Appendix 11 Urinary tract infection diary days 2-8.

## Abbreviations

GP: general practitioner
ICC: intraclass correlation coefficient
ISBAR: Identification, Situation, Background, Assessment, and Recommendation
PI: principal investigator
UTI: urinary tract infection
