# Supplementary figures and images for: Reducing Antibiotic Prescriptions for Urinary Tract Infection in Nursing Homes Using a Complex Tailored Intervention Targeting Nursing Home Staff: Protocol for a Cluster Randomized Controlled Trial
Source: JMIR Res Protoc. 2020 May 8;9(5):e17710. doi: 10.2196/17710 (PMC7244999; doi:10.2196/17710)

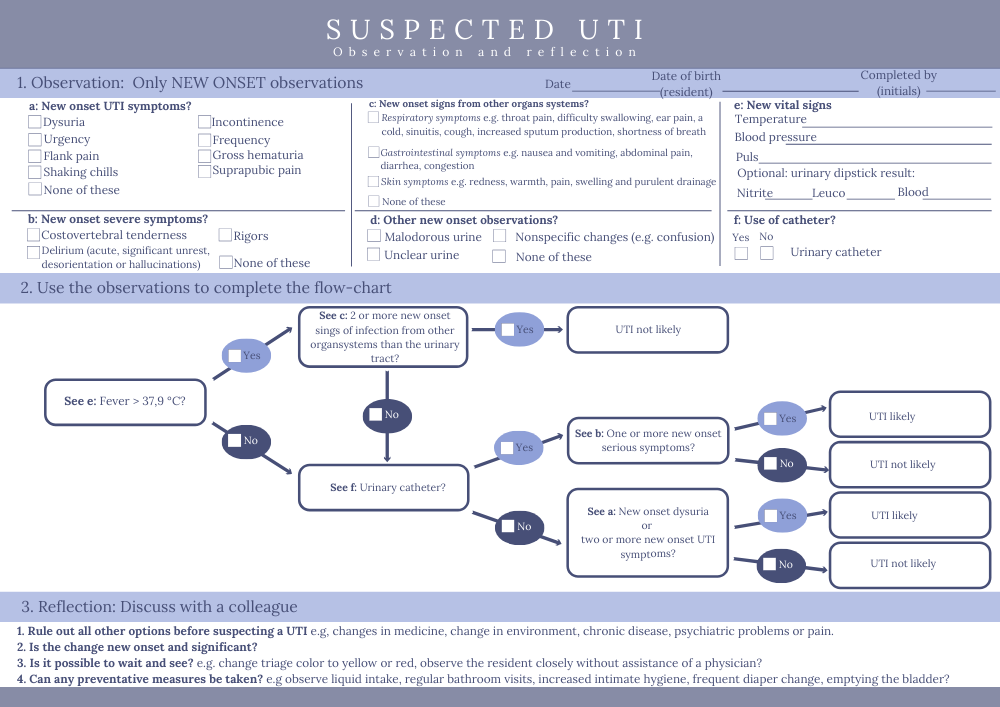

Supplement: Multimedia Appendix 1 [file resprot_v9i5e17710_app1.png]

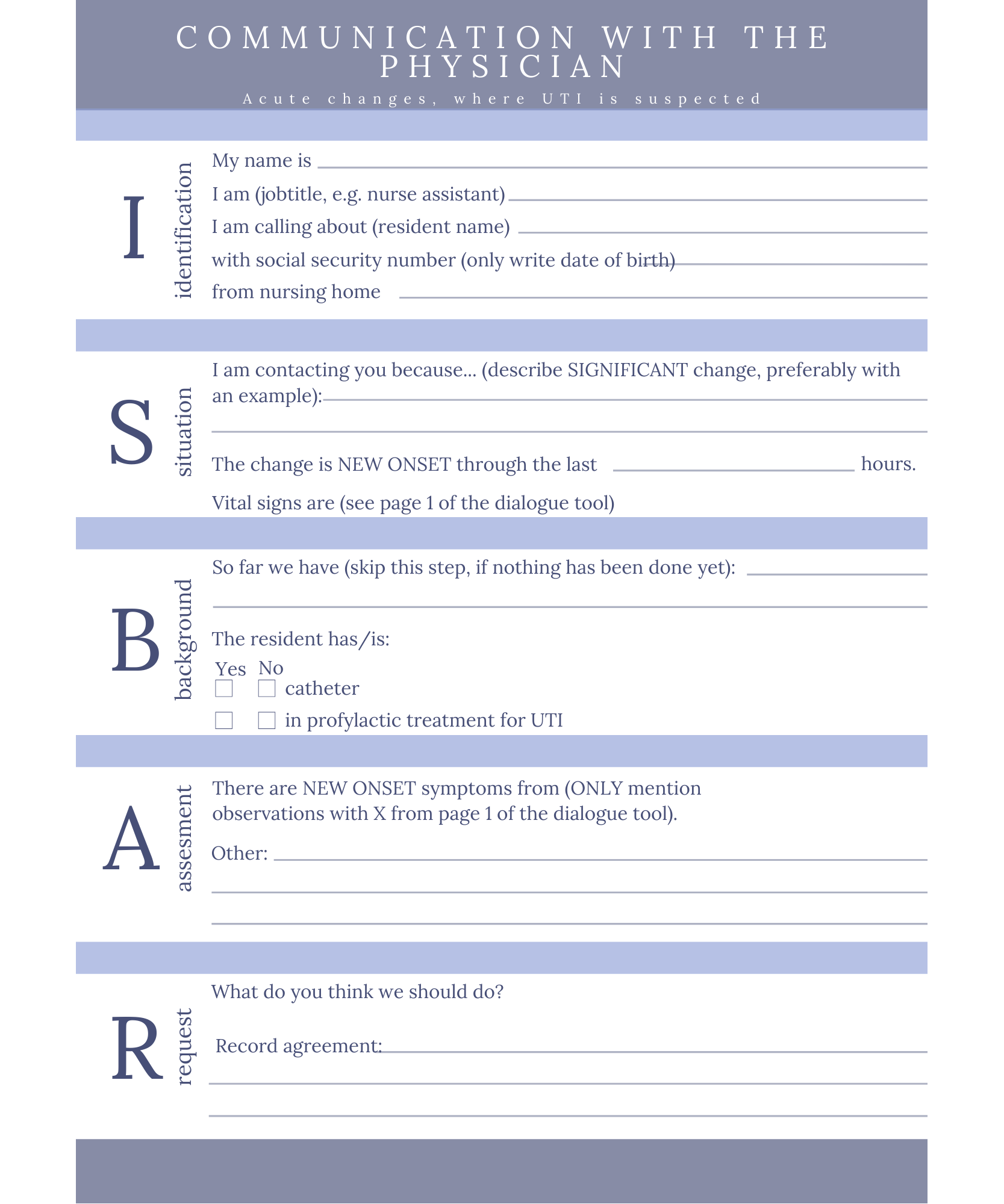

Supplement: Multimedia Appendix 2 [file resprot_v9i5e17710_app2.png]

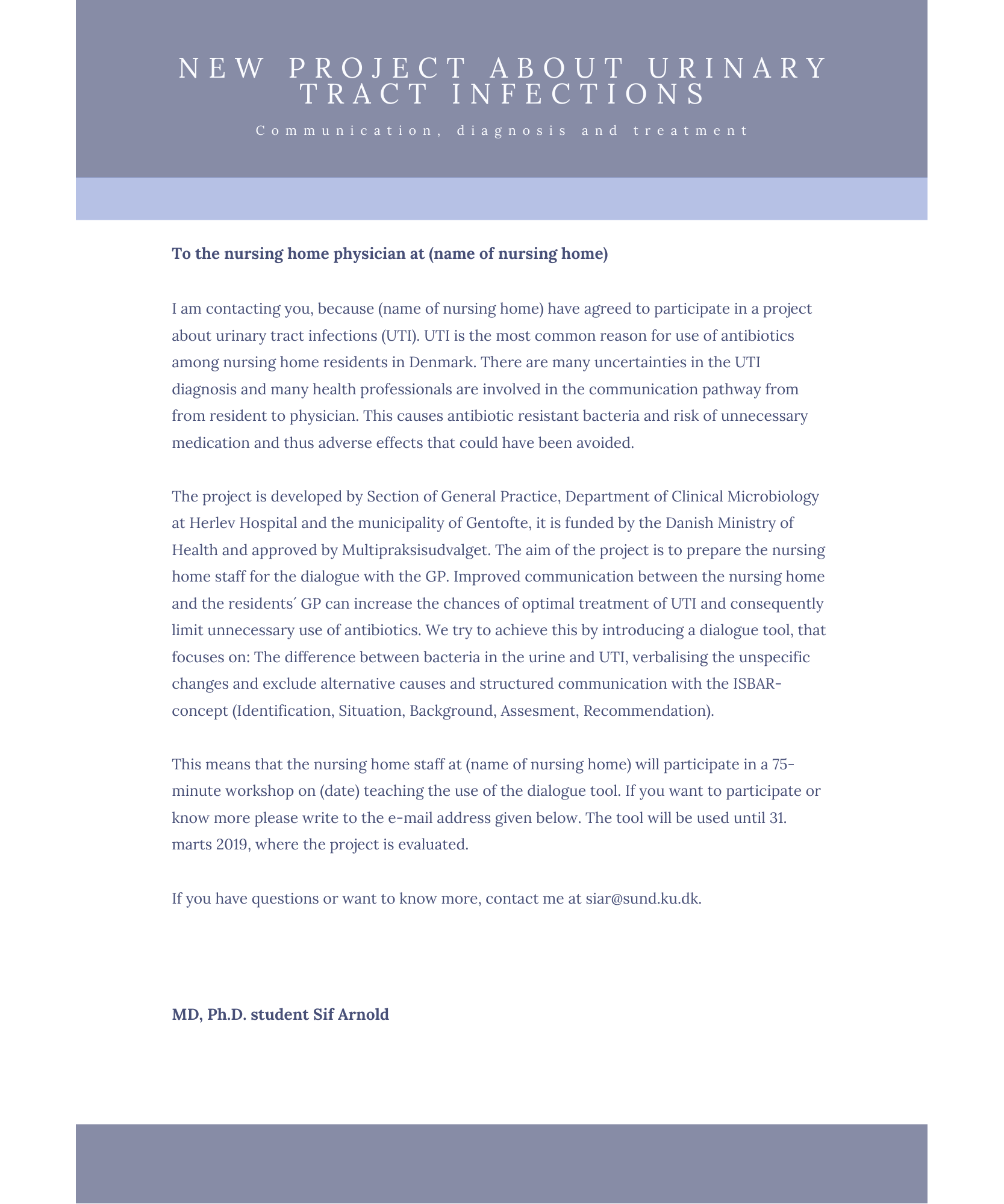

Supplement: Multimedia Appendix 3 [file resprot_v9i5e17710_app3.png]

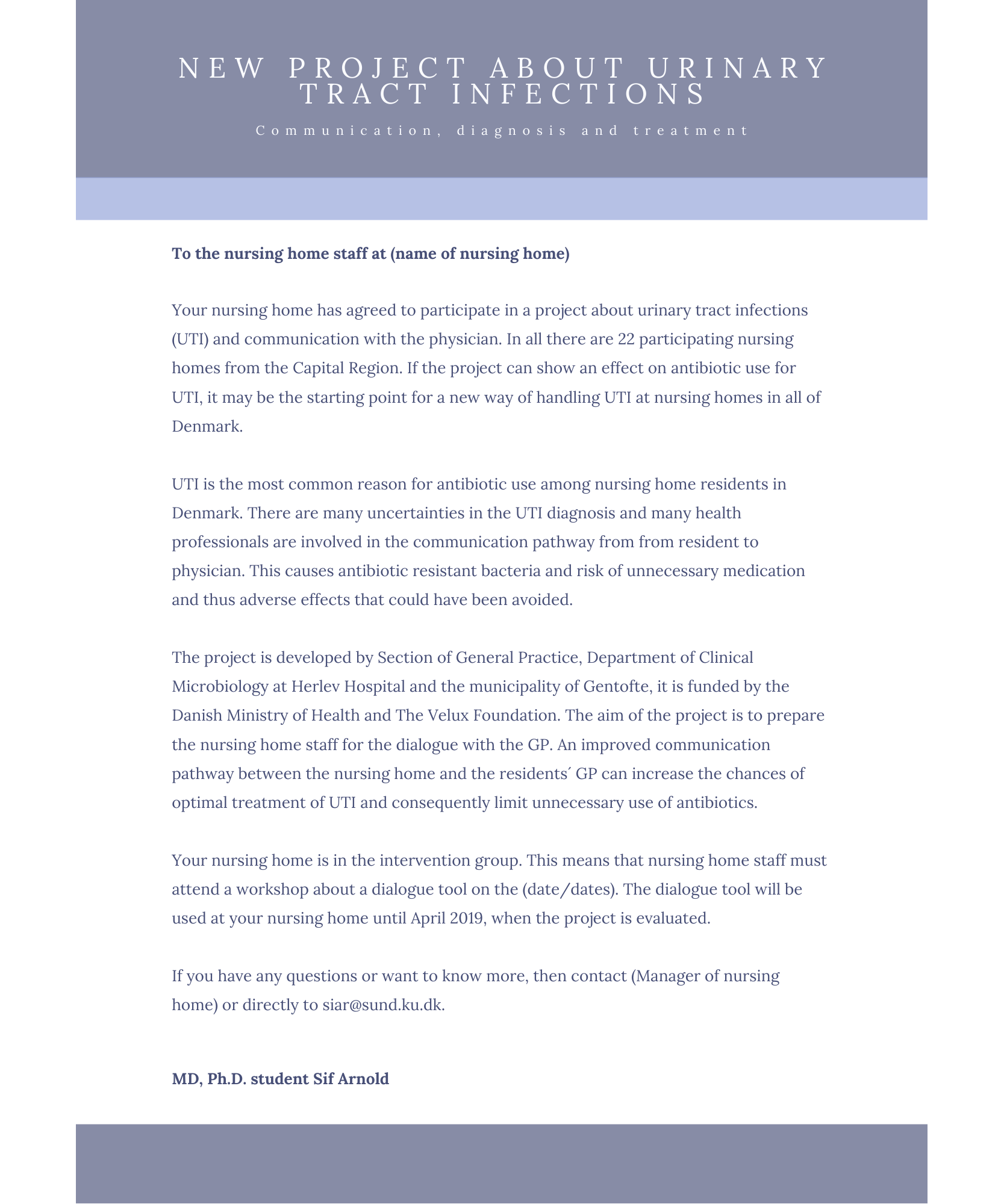

Supplement: Multimedia Appendix 4 [file resprot_v9i5e17710_app4.png]

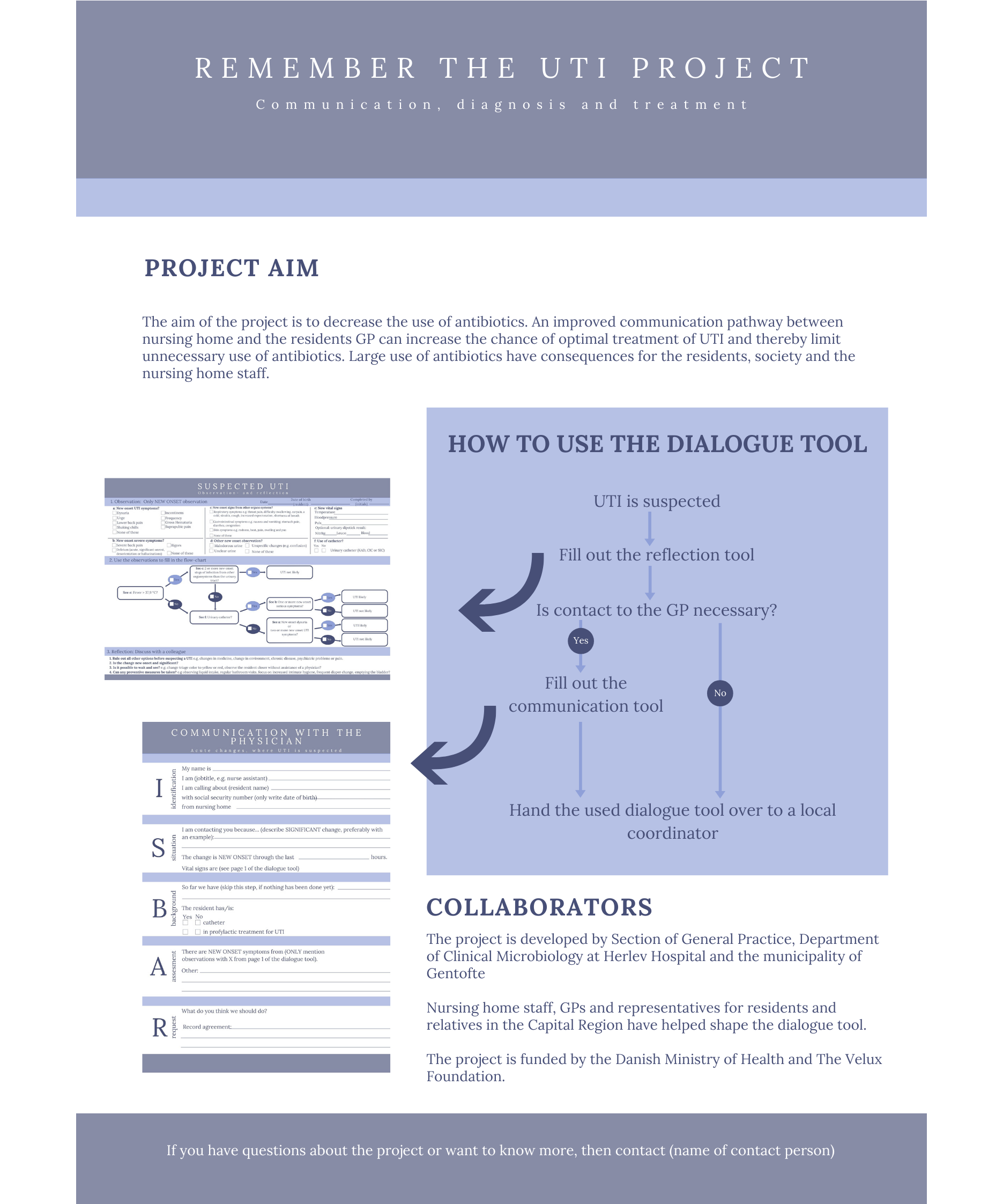

Supplement: Multimedia Appendix 5 [file resprot_v9i5e17710_app5.png]

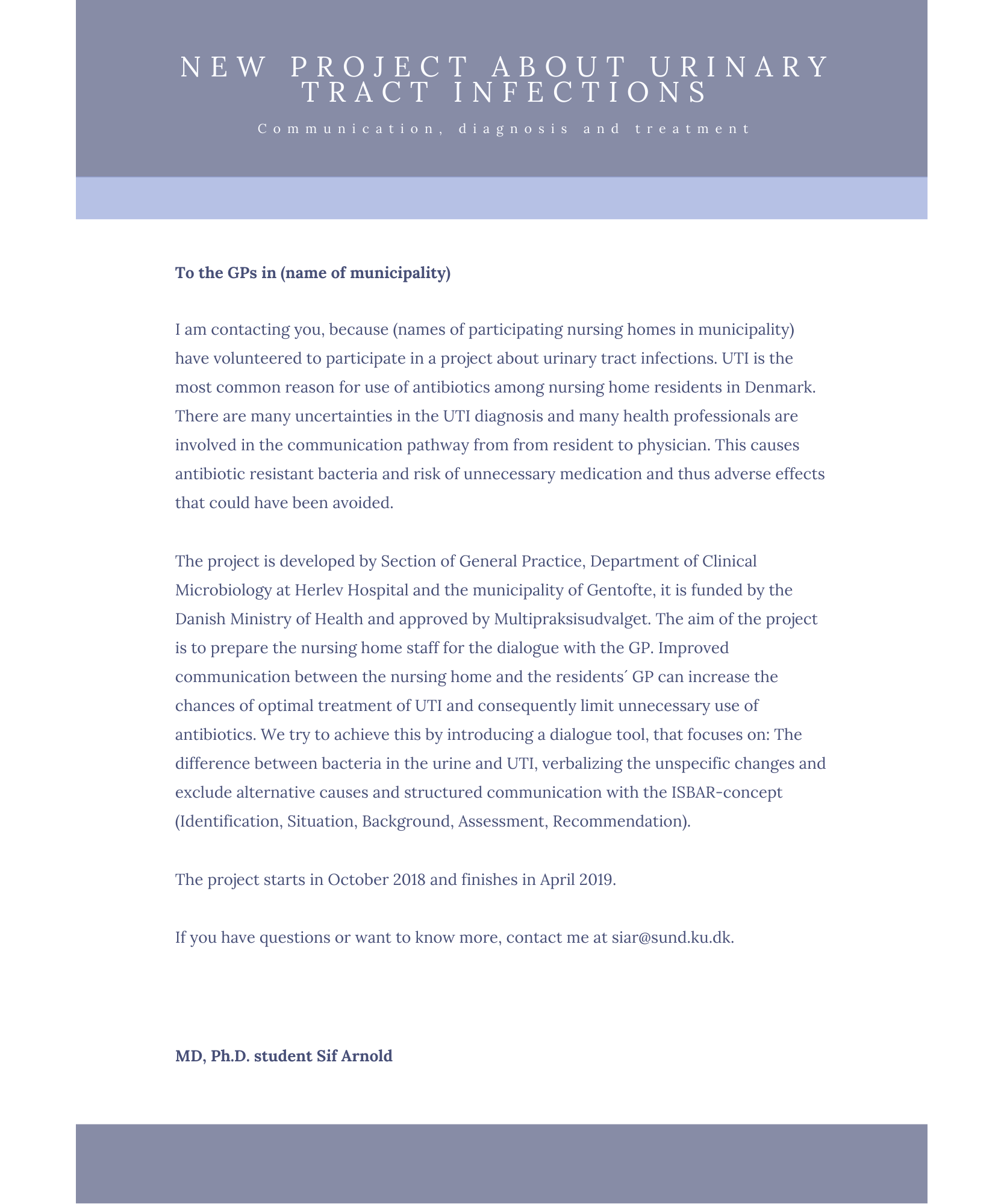

Supplement: Multimedia Appendix 6 [file resprot_v9i5e17710_app6.png]

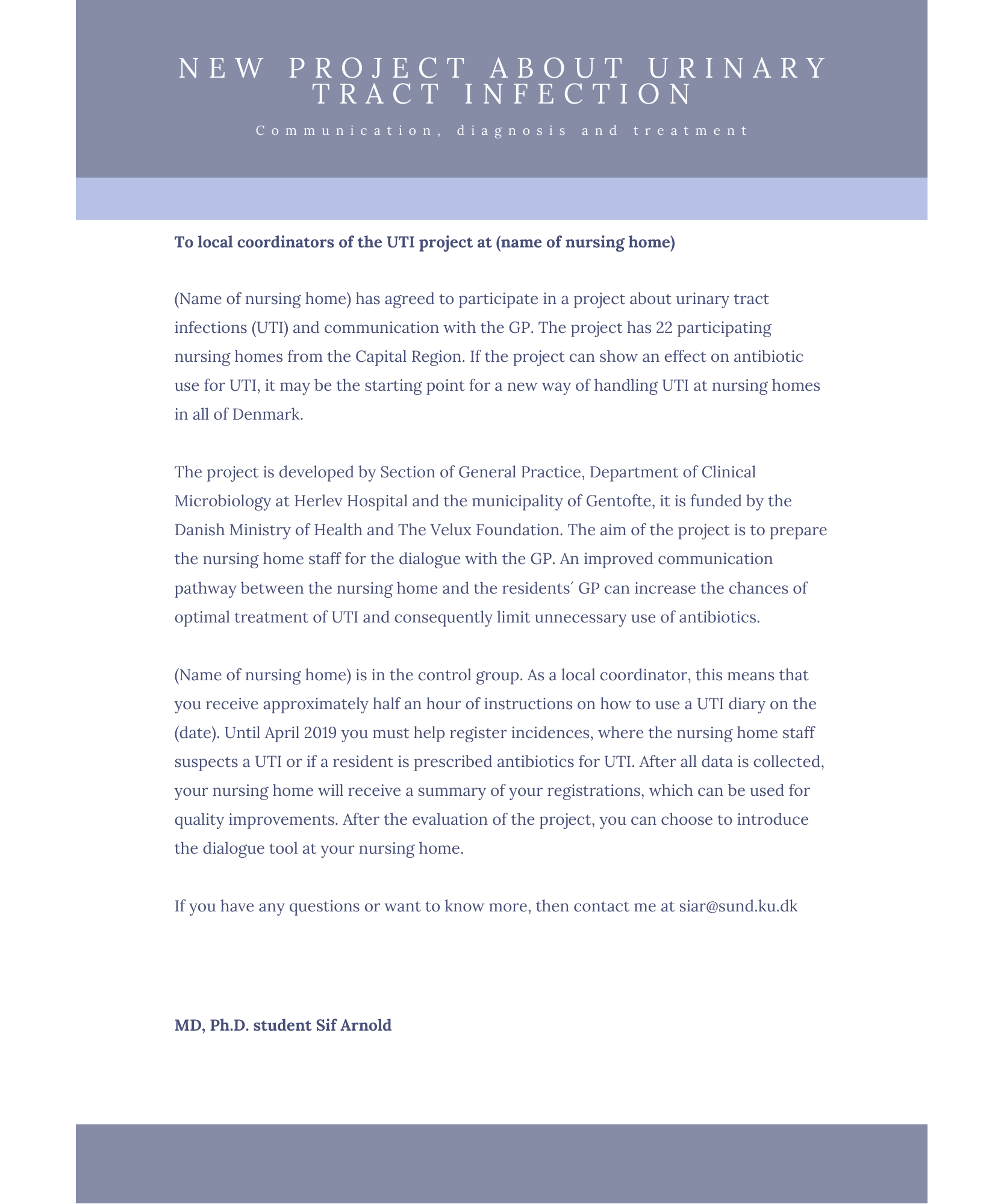

Supplement: Multimedia Appendix 7 [file resprot_v9i5e17710_app7.png]

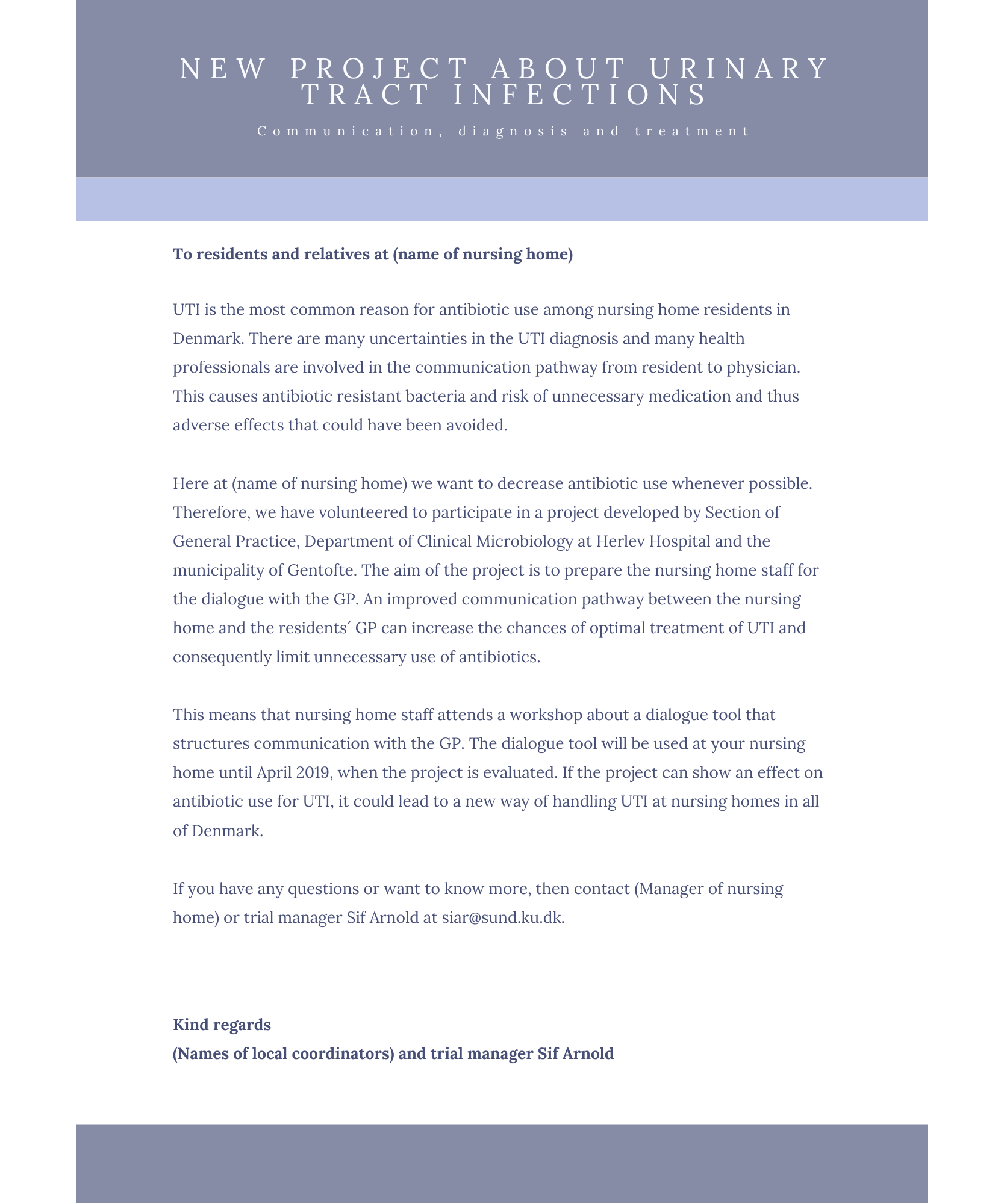

Supplement: Multimedia Appendix 8 [file resprot_v9i5e17710_app8.png]

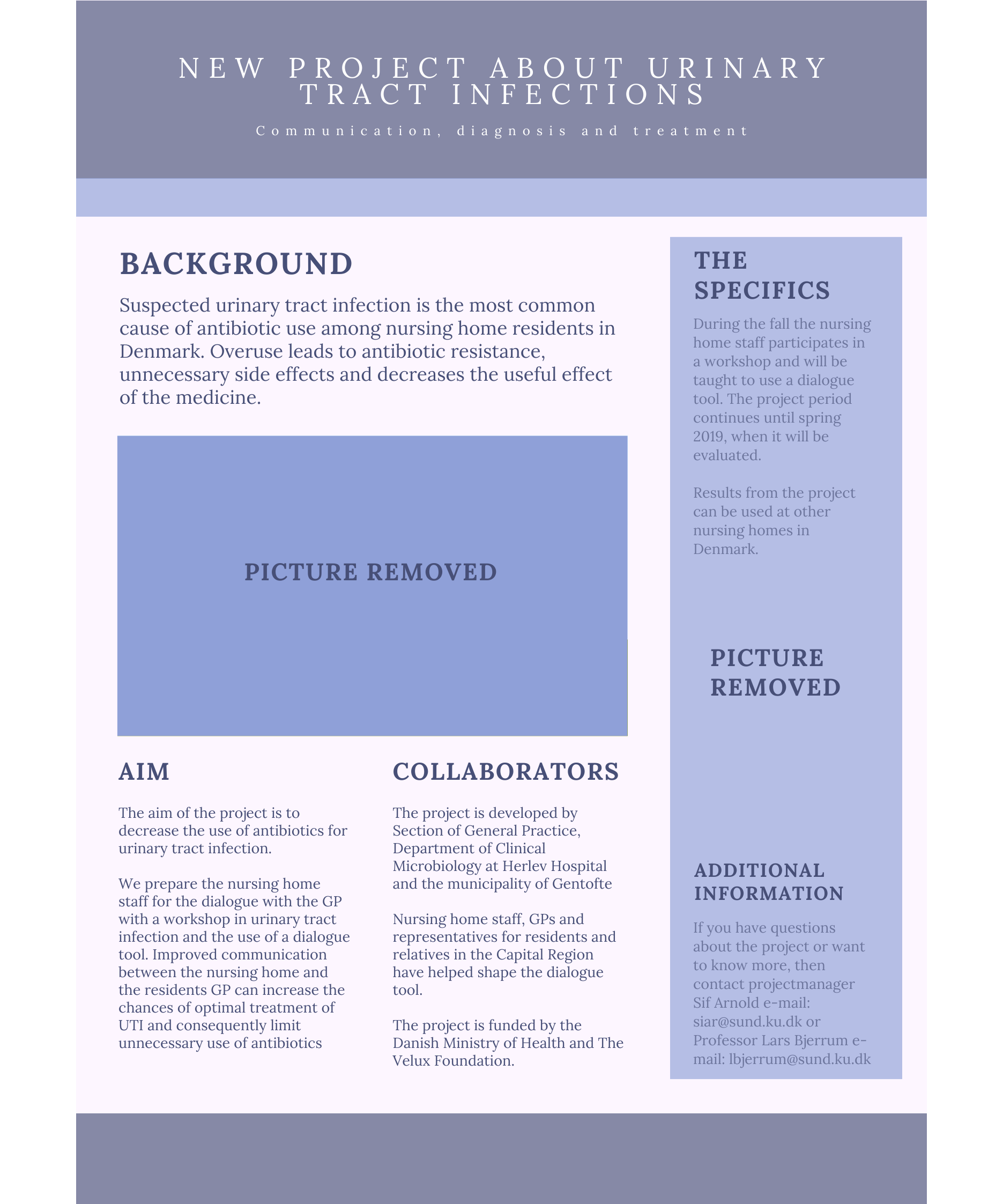

Supplement: Multimedia Appendix 9 [file resprot_v9i5e17710_app9.png]

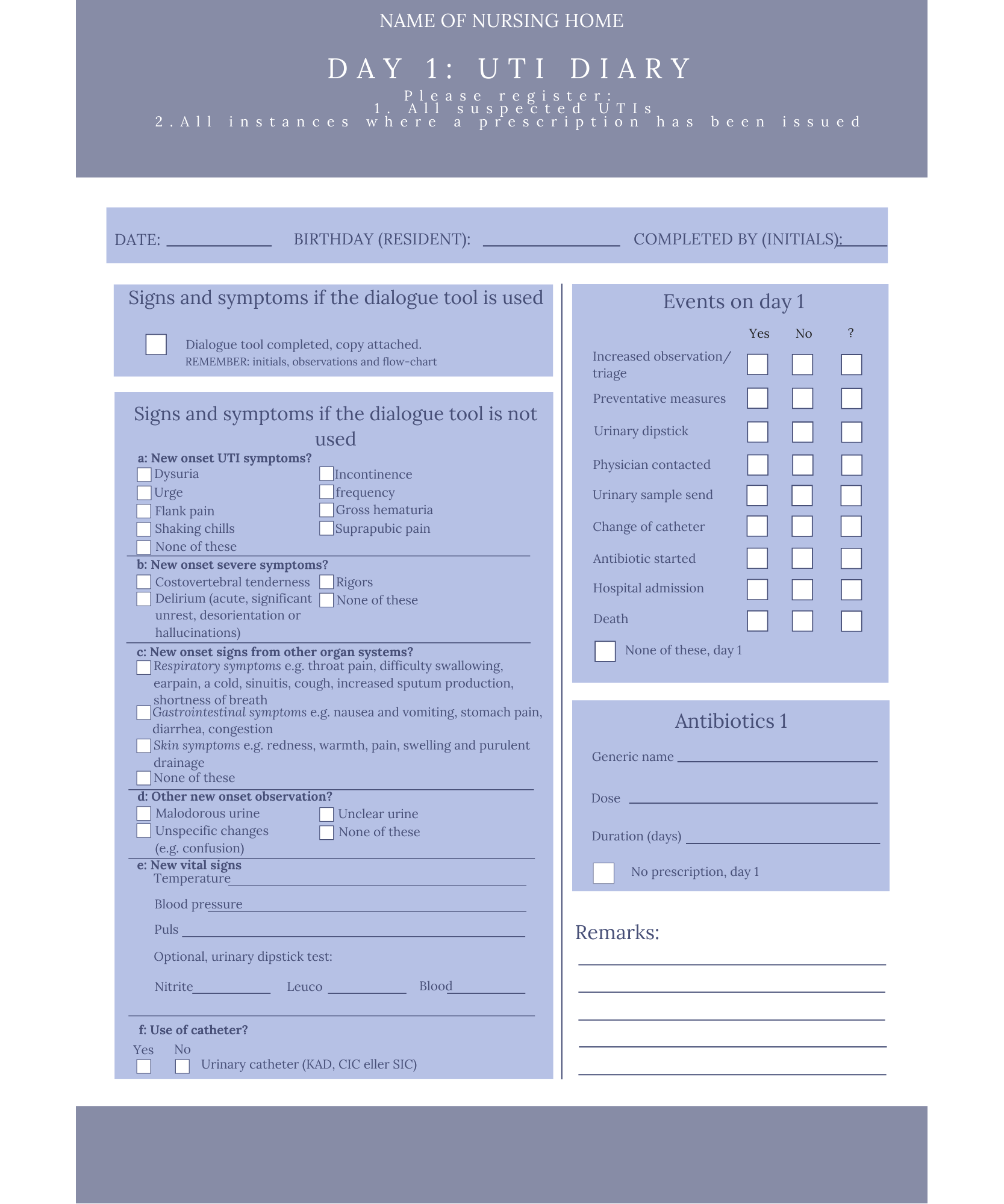

Supplement: Multimedia Appendix 10 [file resprot_v9i5e17710_app10.png]

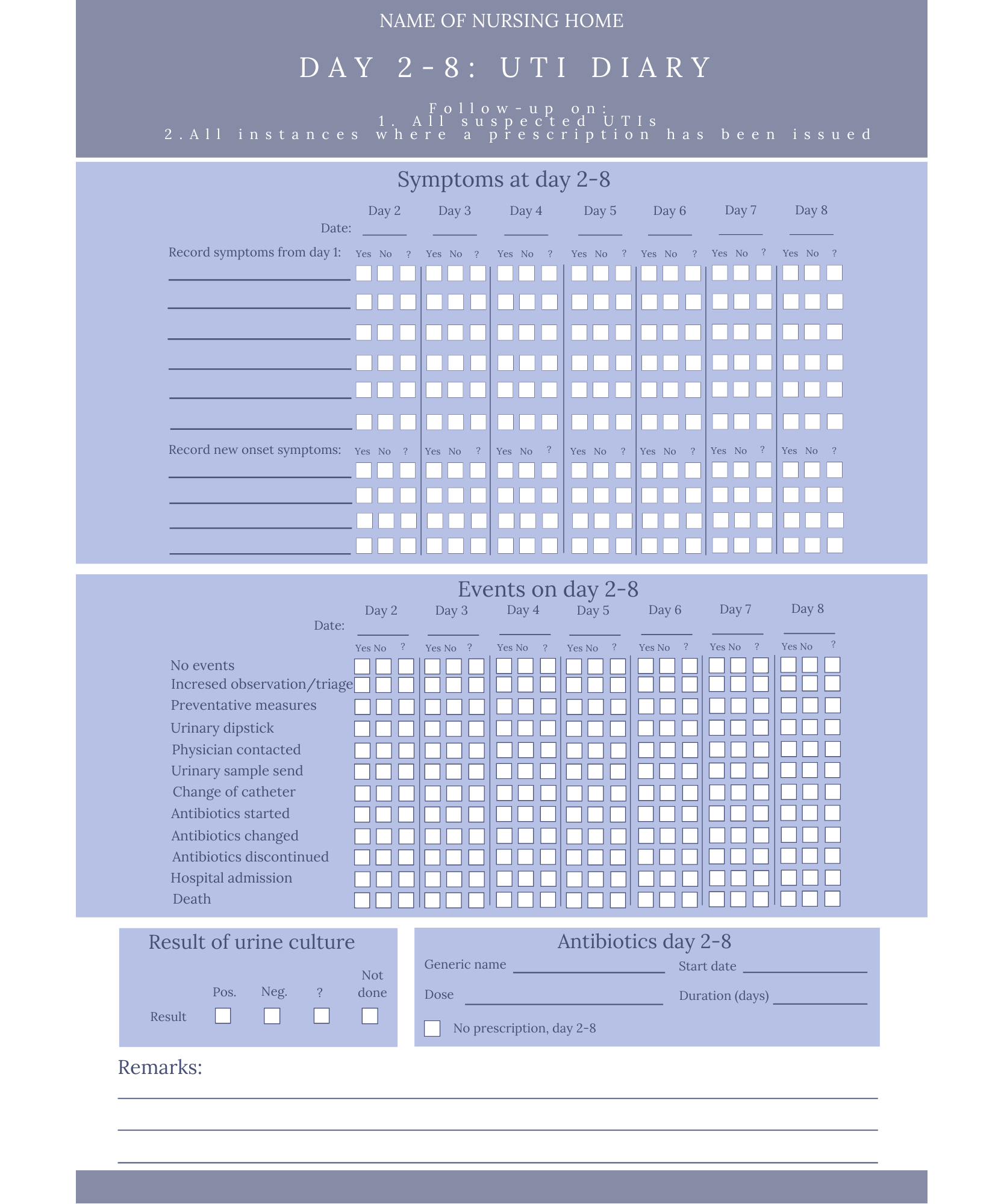

Supplement: Multimedia Appendix 11 [file resprot_v9i5e17710_app11.png]
